# Evaluation of Nanoporous Carbon Synthesized from Direct Carbonization of a Metal–Organic Complex as a Highly Effective Dye Adsorbent and Supercapacitor

**DOI:** 10.3390/nano9040601

**Published:** 2019-04-11

**Authors:** Xiaoze Shi, Shuai Zhang, Xuecheng Chen, Ewa Mijowska

**Affiliations:** 1 Nanomaterials Physicochemistry Department, Faculty of Chemical Technology and Engineering, West Pomeranian University of Technology, Szczecin, Piastów Ave. 42, 71-065 Szczecin, Poland; xiaoze.shi@zut.edu.pl (X.S.); shuai.zhang@zut.edu.pl (S.Z.); 2 State Key Laboratory of Polymer Physics and Chemistry, Changchun Institute of Applied Chemistry, Chinese Academy of Science, Changchun 130021, China

**Keywords:** nanoporous carbon, adsorption properties, dye, adsorption models, supercapacitor

## Abstract

The synthesis of interconnected nanoporous carbon (NPC) material from direct annealing of ultra-small Al-based metal–organic complex (Al-MOC) has been demonstrated. NPC presents a large accessible area of 1054 m^2^/g, through the Methylene Blue (MB) adsorption method, which is comparable to the high specific surface area (SSA) of 1593 m^2^/g, through an N_2_ adsorption/desorption analysis. The adsorption properties and mechanisms were tested by various dye concentrations, pH, and temperature conditions. The high MB accessible area and the good electrical conductivity of the interconnected NPC, led to a large specific capacitance of 205 F/g, with a potential window from 0 to 1.2 V, in a symmetric supercapacitor, and a large energy density of 10.25 Wh/kg, in an aqueous electrolyte, suggesting a large potential in supercapacitors.

## 1. Introduction

Nanoporous carbon (NPC) materials with large specific surface area (SSA), have been applied in different fields, especially, as electrode materials and dye adsorbents [1,2,3]. Dyes are applied in many different industrial fields, such as paper, textile, rubber, food, leather, cosmetics, plastic, and others. However, used dyes also become harmful, due to the fact that they can not only reduce light penetration and photosynthesis in water, but can also contain toxic and carcinogenic chemicals that might be a threat to human health [4]. Unfortunately, most of the wasted dyes are quite stable in the real environment [5]. Therefore, removing dyes from industrial waste water, through efficient technologies, is quite urgent. Adsorption is considered as an economical and efficient method. The adsorption method has the advantages of a simple design, easy operation, and possible regeneration. Methylene Blue (MB) has become a model cationic dye for dye adsorption investigations [6,7]. Various adsorbents, such as biomass materials [8,9,10,11,12], carbon nanotubes [13,14,15,16], graphene-based materials [17,18,19,20], and magnetic materials [21,22], have been investigated in the field of dye removal. Additionally, researchers always use the MB adsorption method to test the surface area of carbon samples, which can indicate the accessible surface of electrodes [23,24].

Carbon materials are also popular as electrode materials, due to their good electrical conductivity, high power density, and good cycling stability [25,26,27,28,29]. Many efforts have been made in these aspects to fabricate NPC materials with excellent properties, including a large SSA and high porosity. As it has been reported, NPC can be synthesized by various methods, such as chemical vapor decomposition (CVD), chemical activation, and template methods [30,31,32]. Among these, the template method has drawn a lot of interest. Easily acquiring zeolites and mesoporous silica have been effectively used as templates, for the formation of NPC with different sizes and structures. Metal–organic frameworks (MOF) have become a new choice of precursors for NPC materials [33,34,35]. There are two types of MOF-derived NPC materials, depending on the production process. One induces a secondary carbon precursor and the other is a direct carbonization of the organic components of MOF. MOF-derived NPC materials possess a high SSA and a tunable pore size. The tunable pores can provide an appropriate size for the electrolyte ions and dye molecules diffusion, thus, increasing the accessible area and providing more physical active sites for the adsorption process. However, the specific capacitance of the MOF-derived NPC reported so far, is still not satisfactory. Due to the large size of the MOF template [36,37], it is difficult for electrolyte and ions to transport into the inner or the center of the micrometer-sized NPC, leaving many inaccessible active sites. Thus, the resultant specific capacitance is limited, especially under a high charging rate. In this respect, decreasing the MOF size is conducive to improving the electrochemical performance. Additionally, a proper pore size distribution (PSD) can also help to improve the capacitance [38].

Herein, we have prepared an interconnected NPC material from a direct carbonization of the ultra-small Al-MOC. NPC presents a high Brunauer-Emmett-Teller (BET) area, showing an excellent adsorption capacity for MB. NPC can adsorb MB with a maximum value of 415 mg/g, via a physical adsorption process, which suggests a large molecular accessible area of 1054 m^2^/g, very close to the SSA from the N_2_ adsorption/desorption analysis (1593 m^2^/g). The large accessible surface area, together with the suitable pore distribution (micropores and mesopores) and high conductivity of the NPC, makes it a good candidate for an electrode material. Hence, a two-electrode supercapacitor has been designed with the NPC material, exhibiting a large specific capacitance of 226 F/g and an energy density of 10.25 Wh/kg, in an aqueous electrolyte.

## 2. Materials and Methods 

### 2.1. Synthesis of NPC 

NPC was fabricated from Al-MOC, according to our previous report [2]. In brief, 2 g of the Al-MOC was heated at 950 °C, for 3 h, in Ar atmosphere and then treated with 10 mL 17% HCl, to remove the metal components. The final product was dried at 105 °C and named as NPC. All the chemicals were purchased from MERCK (Darmstadt, Germany).

### 2.2. Characterization

Transmission electron microscopy (TEM), High-resolution transmission electron microscopy (HRTEM) (Tecnai F30, FEI, Eindhoven, The Netherlands), Scanning Electron Microscopy (SEM, Hitachi SU8020, Tokyo, Japan), X-ray diffraction using Copper K-α with an X-ray wavelength of 1.5406 Å (XRD, X’Pert PRO Philips diffractometer, Almelo, Holland), and Raman scattering (Renishawmicro, Renishaw, London, UK; λ = 785 nm) were performed, to characterize the structural properties. Thermogravimetric analysis (TGA, DTA-Q600 SDT TA, New Castel, DE, USA) was conducted to determine the composition. The N_2_ adsorption/desorption analysis (Micromeritics ASAP 2010M, Boynton Beach, FL, USA) were used to calculate the SSA and the PSD. UV-vis spectrophotometer (Thermo Scientific, Waltham, MA, USA) was applied to calculate the concentration of MB.

### 2.3. Adsorption Equilibrium Isotherm

NPC (W: 0.02 g) was dissolved in a constant volume (V: 0.02 L) of MB aqueous solutions, with different initial concentrations (*C*_0_: 100 to 900 mg/L), at 25 °C, for 24 h. The MB concentrations in the supernatant were measured as *C*_e_ (mg/L) and the adsorbed dye amounts by NPC (*q*_e_, mg/g) were calculated as follows:

(1) $$q_{e}=\frac{\left(C_{0}-C_{e}\right)V}{W}$$

### 2.4. Adsorption Kinetics

The adsorption kinetic measurements were carried out by continuously stirring the NPC and MB solutions (*C*_0_: 200, 400, or 600 mg/L). The concentrations of MB (*C*_t_: mg/L) in the supernatant were checked at preset time intervals (*t*: min), to calculate the adsorbed MB amount at time (*q*_t_, mg/g):

(2) $$q_{t}=\frac{\left(C_{0}-C_{t}\right)V}{W}$$

### 2.5. Adsorption Thermodynamics

To observe the influence of different temperatures on the adsorption capacity, NPC and MB solutions (500 or 700 mg/L) were placed under different temperatures (25, 35, 45, or 55 °C) and stirred for 24 h. The concentrations of MB and the amounts of MB adsorbed onto the NPC, at equilibrium, were measured, similar to that of adsorption equilibrium experiments.

### 2.6. Effect of pH Values

Five different pH values (3.62, 5.14, 7.21, 8.76, and 10.20) were investigated by adjusting the pH of MB (400 mg/L) solution, with NaOH or HCl (0.1 M). 

### 2.7. Electrochemical Evaluation

NPC (80 wt.%) was mixed with carbon nanotubes (Sigma, Kawasaki, Kanagawa Prefecture, Japan; 10 wt.%) and polyvinylidenedifluoride (Solvay, Brussels, Belgium; 10 wt.%), and the electrode (*d*: 1 cm) was prepared by pressing the mixture at 10 MPa. A two-electrode system was used with active materials mass of 2 mg. The cyclic voltammetry (CV), galvanostatic charging/discharging (GCD), and electrochemical impedance spectroscopy (EIS) tests were performed on the EC-LAB VMP3 workstation (BioLogic Science Instruments, Seyssinet-Pariset, France), with 1 M Li_2_SO_4_ aqueous solution.

## 3. Results and Discussion

**Figure 1:**
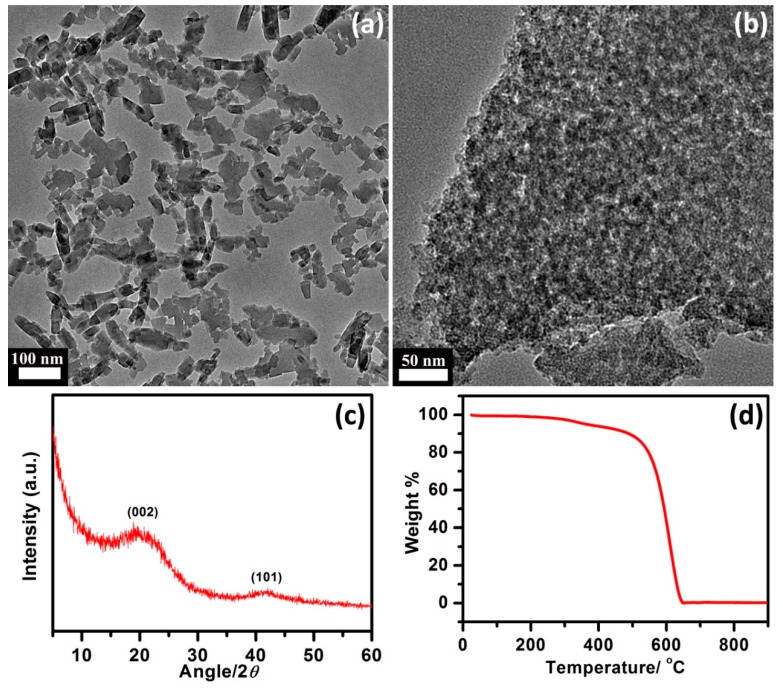
TEM images of Al-based metal–organic complex (Al-MOC) (**a**) and nanoporous carbon (NPC) (**b**); XRD pattern (**c**) and TGA curve (**d**) of NPC.

The morphology of NPC material is presented by SEM and TEM. Figure 1 shows the TEM images of the Al-MOC, before (Figure 1a) and after carbonization (Figure 1b). Al-MOC, which had an XRD pattern (Figure S1) comparable to the MIL-53 (Al) from the report [39], exhibited separated nanocubes with ultra-small size around 100 nm, while NPC turned into an interconnected three-dimensional bulk structure, after sintering under high temperatures (Figure 1b). SEM image of NPC also indicated a large size, after annealing (Figure S2). XRD pattern of the obtained NPC is shown in Figure 1c. The NPC powder presents the graphitic carbon (002) and (101) diffractions. The presence of (002) diffraction at 2θ = 23° shifted to the left, compared to the perfect graphite diffraction at 2θ = 26°, suggesting that the NPC sample had a low crystallinity [40]. TGA data (Figure 1d) showed a negligible weight of the remaining NPC, after heating to 900 °C, suggesting a successful removal of alumina.

**Figure 2:**
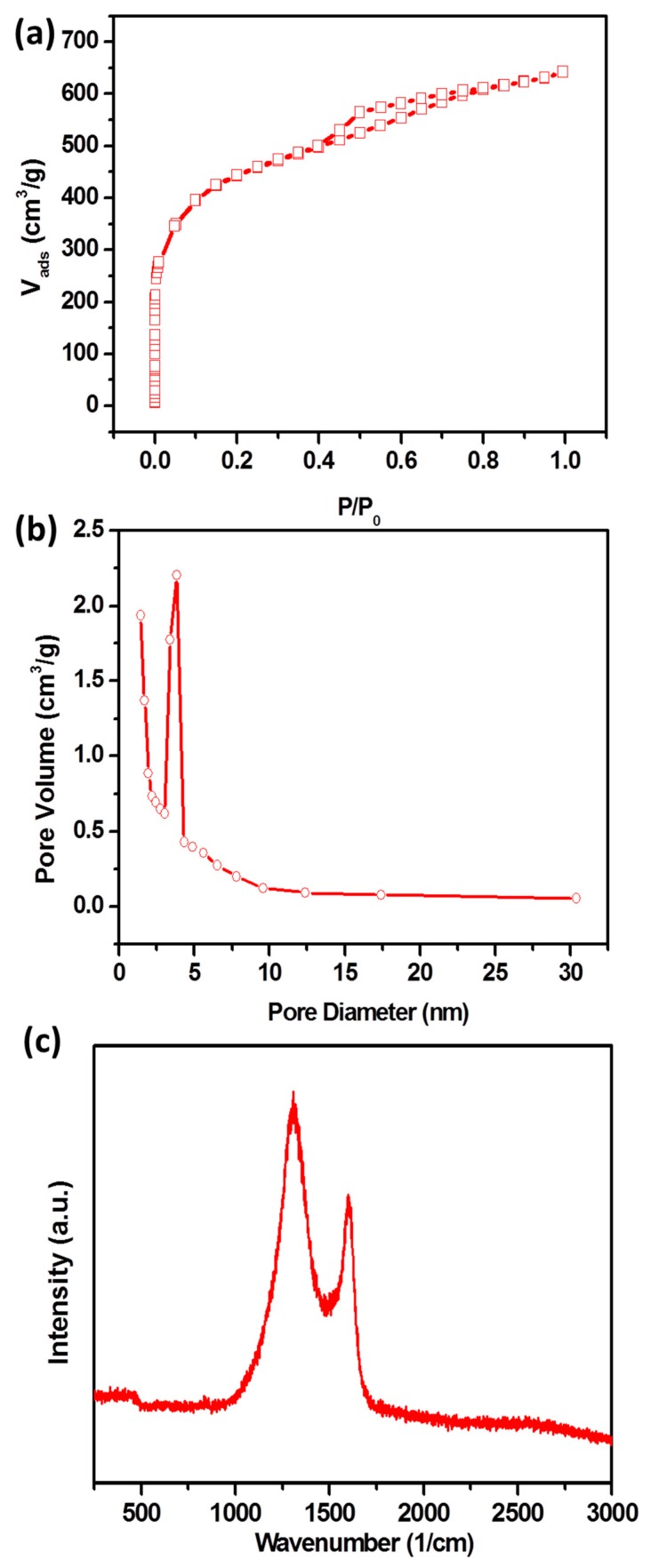
Nitrogen adsorption/desorption isotherms (**a**), pore size distributions (**b**), and Raman spectrum (**c**) of NPC.

Although NPC showed an interconnected structure after high-temperature annealing, the HRTEM (Figure S3) images still exhibited an amorphous structure. Notably, irregular mesopores can be found from the agglomerate (Figure S3a–c). In order to achieve the detailed information, the N_2_ adsorption–desorption isotherm within the relative pressure of 0–1 was measured. The isotherm in Figure 2a exhibits a typical type IV profile, suggesting the existence of micro-, meso- and macropores. It was calculated that the SSA of NPC was 1593 m^2^/g and the total pore volume was 2.49 cm^3^/g (Figure 2b), due to the new pores that appeared among the agglomerated complex in bulk NPC. Raman spectrum (Figure 2c) of the NPC sample showed two peaks at 1324 (D band) and 1580 1/cm (G band). The D and G bands were ascribed to the disordered carbons and ideal graphitic carbon, respectively [41]. The intensity ratio I_D_/I_G_ for the NPC was 1.36, suggesting the disordered nature for NPC.

**Figure 3:**
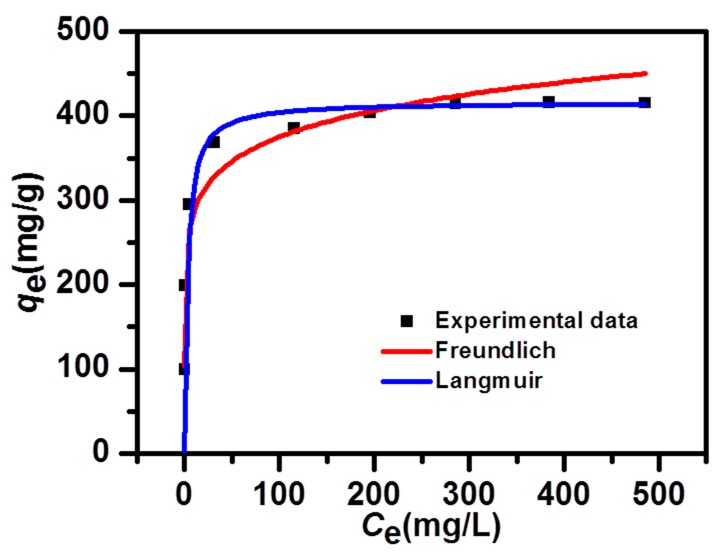
Non-linear fits of the Langmuir and Freundlich isotherm models, to the Methylene Blue (MB) adsorption.

Encouraged by the large SSA and the well-distributed porous structure, the adsorption properties of NPC were tested. First, the adsorption process was studied with different dye concentrations. A fast color fading could be observed with the low-concentration groups, and then the adsorption equilibrium, proceed slowly with the high-concentration groups. The fast color fading indicated a fast adsorption rate, which was contributed by the large SSA, as well as the large pore volume. Figure 3 shows that there was a significant increase of the adsorption capacities, with the first four concentrations, which grew slowly, with the latter five concentrations. This could be proved by the well-fitted Langmuir model [42], indicating a monolayer adsorption behavior, which meant that no further dye adsorption could happen at the occupied site of the NPC. The fitted Langmuir isotherm could be defined in the following form:

(3) $$\frac{C_{e}}{q_{e}}=\frac{C_{e}}{q_{\max}}+\frac{1}{K_{L}q_{\max}}$$

**Figure 4:**
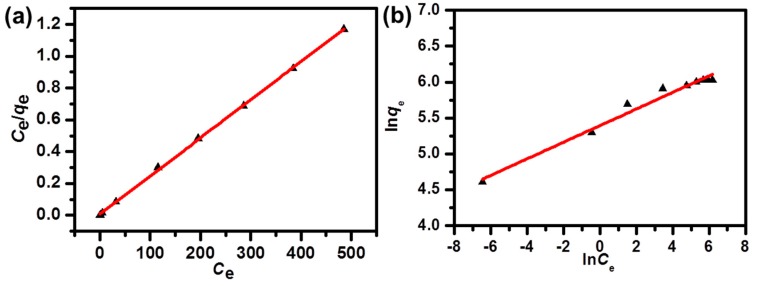
Linear fits of the Langmuir (**a**) and Freundlich (**b**) isotherms for the MB adsorption on the NPC samples.

From Figure 4a, *q*_max_ (mg/g) is the maximum amount of MB adsorbed onto the NPC and the constant (*K*_L_: L/mg) had a relationship to the rate of adsorption. Additionally, the *R*_L_ parameter which indicated the adsorption process to be favorable (0 < *R*_L_ < 1) or unfavorable with the isotherm (*R*_L_ > 1), was calculated as follows [43]:

(4) $$R_{L}=\frac{1}{1+K_{L}C_{0}}$$

The Freundlich isotherm model which demonstrated a heterogeneous adsorption process [44], could be written in the following form:

(5) $$\ln q_{e\;}=\ln K_{F}+\frac{1}{n}\ln C_{e}$$

where the constants (*K*_F_ and *n*) are related to the intercept and the slope of Figure 4b. The parameters for adsorption isotherms calculated from the above Equations (3)–(5) are shown in Table 1. The correlation coefficient (*R*^2^) values indicated that the dye adsorption process was well-matched to both models, but was better matched to the Langmuir isotherm model. It revealed that the dye adsorption started on both the homogeneous and heterogeneous active sites of NPC. The heterogeneous sites were from the possible functional groups contained on the NPC surface. There were negligible differences between the maximum adsorption capacity (*q*_max_) fitted by the Langmuir model (417 mg/g), and the experimental value (415 mg/g), which also proved to be a better match to the Langmuir isotherm. According to Table 1, the *R*_L_ value was near zero (0.0035), illustrating that the adsorption process was favorable and irreversible. Value *n* (8.656) in the range of 1–10, also illustrated that this adsorption was favorable [45,46].

**Figure 5:**
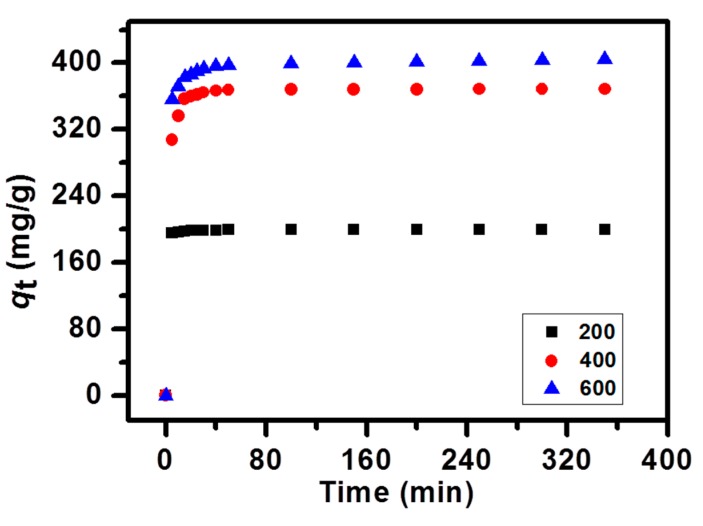
Effect of contact time on the adsorption capacity of MB on NPC samples.

To study the effect of time, adsorption kinetics was measured by choosing different time intervals. Adsorption kinetics could also help to quantify the adsorption rate and illustrate the mechanism of adsorption. The adsorption kinetics of the MB-NPC was obtained by three different MB concentrations (200, 400, and 600 mg/L). The MB adsorption was rapid in the first 5 min (Figure 5), and then the adsorption rate gradually slowed down, with the proceeding time. At last, the adsorption achieved equilibrium, within 2 h. Four kinetics models were applied to figure out the kinetic mechanism of the dye adsorption. First, the adsorption kinetics was examined by the pseudo-first-order model. The equation is described as follows [47]:

(6) $$\ln\left(q_{e}-q_{t}\right)=\ln q_{e}-k_{1}t$$

**Figure 6:**
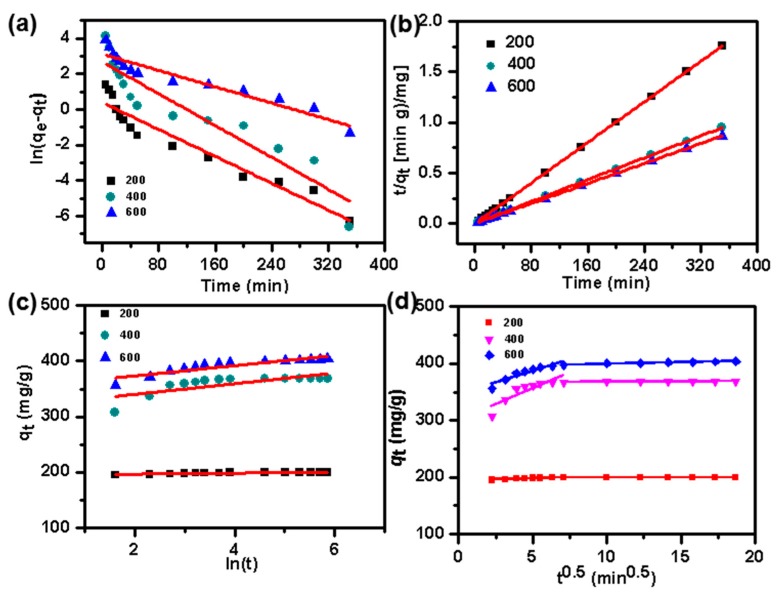
Linear fits of pseudo-first-order kinetics (**a**), pseudo-second-order kinetics (**b**), Elovich kinetic (**c**), and intraparticle diffusion (**d**) models for the adsorption of MB on the NPC samples.

Based on the equation, the plots of ln(*q*_e_ *− q*_t_) versus t (Figure 6a) can be used to calculate *k*_1_ and *q*_e_. Second, the pseudo-second-order equation can be expressed as follows [48]:

(7) $$\frac{t}{q_{t}}=\frac{1}{k_{2}q_{e}^{2}}+\frac{1}{q_{e}}t$$

Based on this equation, *q*_e_ and *k*_2_ calculated from the plots of t/*q*_t_ versus t (Figure 6b) are shown in Table 2. The corresponding *R*^2^ values in the pseudo-first-order kinetic model (<0,920) are relatively smaller than in the pseudo-second-order kinetic model (0.999). Moreover, the *q*_e_ values of the pseudo-second-order model from the fitted linear plots, are better agreed with the experimental data than those of the pseudo-first-order model, indicating that, in this work, it was more appropriate using the pseudo-second-order kinetic model, to describe the adsorption kinetics.

The Elovich model, which can be used to identify chemical adsorption, is described as follows [49]:

(8) $$q_{t}=\frac{1}{\beta }\ln\left(\alpha \beta \right)+\frac{1}{\beta }\ln\left(t\right)$$

where *α* and *β* values correspond to the initial adsorption and desorption rate, respectively. As shown in Table 2, the large *α* shows the viability of the MB-NPC adsorption, while the low value of *β* confirms that the MB-NPC adsorption is essentially irreversible [50,51]. This model, with a low value of *R*^2^, has a poor linearity (Figure 6c), considering that the mechanism of adsorption did not occur via chemical adsorption.

Dye adsorption might involve the following three sequential steps—(1) external adsorption on the sorbent surface, (2) dye diffusion to the sorbent pore or intraparticle diffusion, and (3) chemical adsorption [7,52,53]. These steps can be investigated by the intraparticle diffusion model described as follows [54]:

(9) $$q_{t=k_{i}t^{0.5}+C}$$

Based on this equation, the plot of *q*_t_ versus t^0.5^ could be used to calculate the intraparticle diffusion rate constant (*k*_i_: mg/g/min^0.5^) and *C* (mg/g) (Figure 6d). The corresponding parameters are listed in Table 2. As shown in Figure 6d, there are two linear plots, by fitting *q*_t_ versus t^0.5^, suggesting that intraparticle diffusion was involved but was not the predominant mechanism in the adsorption process. Thus, there might be other factors that could influence the adsorption kinetics [53,55]. The concentrations showed two defined stages (Figure 6d). For all concentrations, the first stage had a higher slope, indicating the external and boundary diffusion of the dye adsorption on the NPC surface. The second linear stage showed a drop in the slope, which could be assigned to the intraparticle diffusion. The C values could be used to evaluate the diffusion resistance, suggesting the thickness of the boundary layer. The C values showed an increasing trend towards the dye concentrations (Table 2), which meant that the boundary layer diffusion had a larger effect on the high initial dye concentration.

**Figure 7:**
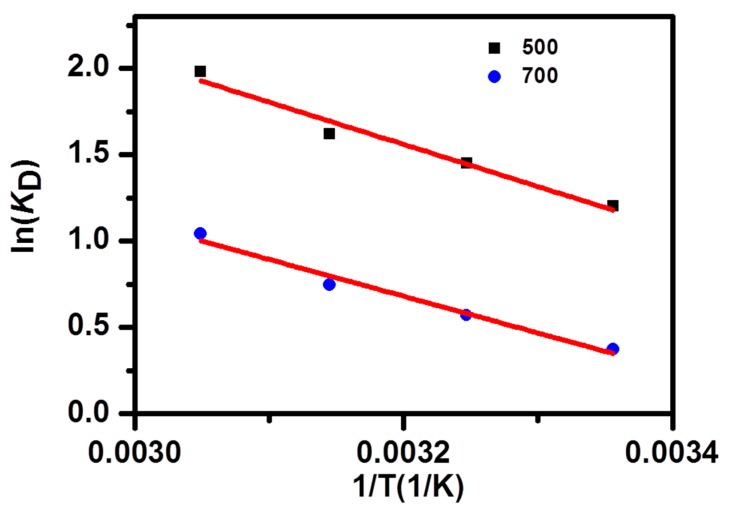
The plot of ln*K*_D_ versus 1/T for the adsorption of MB on the NPC samples.

Temperature is another important factor for the dye adsorption. The temperature effect was investigated at 25, 35, 45, and 55 °C, with two MB initial concentrations (500 or 700 mg/L). The thermodynamic equations were as follows:

(10) $$K_{D}=\frac{q_{e}}{C_{e}}$$

(11) $$\Delta G=-\mathrm{RTln}K_{D}$$

(12) $$\ln K_{D}=\frac{\Delta S}{R}-\frac{\Delta H}{\mathrm{RT}}$$

where *K*_D_ was the adsorption constant, T (K) is the temperature, *R* (8.314 J/mol/K) is the gas constant, and ΔG^θ^ is the change of the Gibbs free energy (kJ/mol), the change of enthalpy ((ΔH^θ^, kJ/mol), and the change of entropy (ΔS^θ^, J/mol/K), which could be calculated from the slope and the intercept of ln*K*_D_ against the 1/T plot (Figure 7). Table 3 shows the thermodynamic parameters obtained from MB-NPC adsorption. The ΔG^θ^ was negative, proving that the adsorption of MB on the NPC was spontaneous in the temperature set, and the positive values of ΔH^θ^ confirmed that the adsorption process was endothermic. Furthermore, ΔG^θ^ changed to more negative values with increasing temperature, indicating that the MB-NPC adsorption was more favorable at a higher temperature, which could also be revealed by the increased experimental adsorption, under a higher temperature. In addition, the values of ΔG^θ^, within the range of −20–0 kJ/mol, indicated that the mechanism of the adsorption process was mainly physical adsorption. The ΔS^θ^ was positive, reflecting the randomness at the solid–liquid interface, during the increasing MB-NPC adsorption.

**Figure 8:**
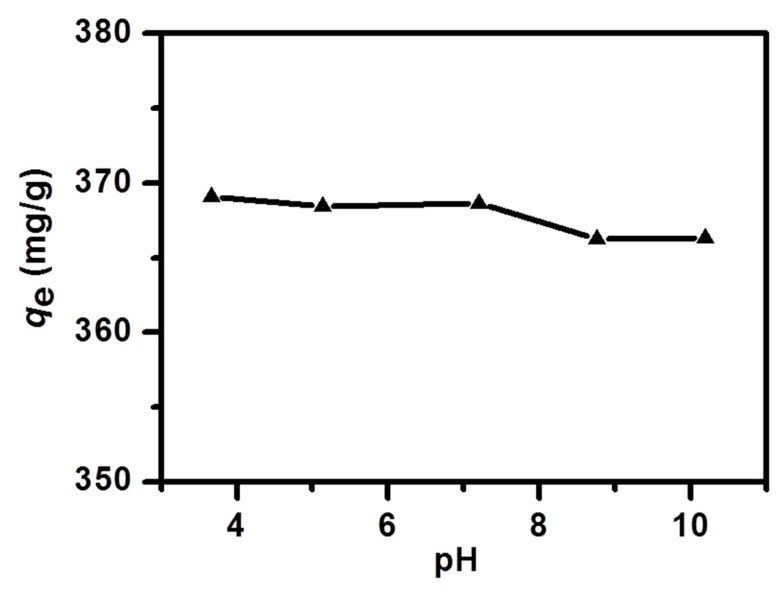
Influence of the solution’s initial pH on the MB adsorption onto the NPC.

Different pH values of the solution were considered to be another factor in the MB-NPC adsorption. As the pH values of the wasted water were various in practice, it was necessary to evaluate the effect of the pH values on the dye removal technologies. The pH values could affect the adsorption capacity because they could remarkably change the surface charge of the adsorbent. They could also influence the electrostatic interactions and chemical reaction between adsorbates and the adsorbent on active sites [56]. In this study, the pH values were changed from 4 to 10 (Figure 8). The adsorption capacity showed a negligible change within the entire pH region, indicating that the adsorption capacity of the NPC with MB, was not influenced by this factor.

**Figure 9:**
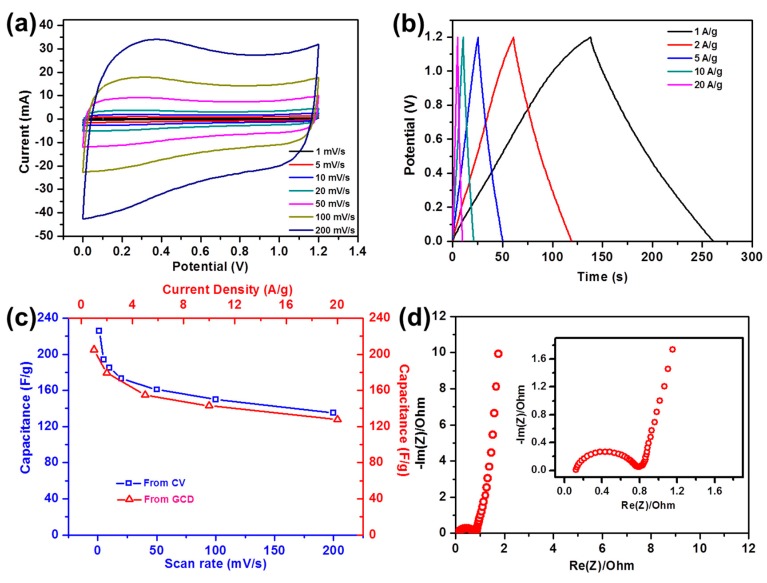
Cyclic voltammetry (CV) curves (**a**) and galvanostatic charging/discharging (GCD) curves (**b**) of NPC; Variations of the specific capacitances (**c**) of NPC from CV and GCD; Nyquist plots (**d**) of NPC (inset shows the Nyquist plots in a high-frequency region).

The electrochemical properties were studied in a full-cell setup, to evaluate the capacitive performance of NPC, in practice. Using 1 M Li_2_SO_4_ aqueous solution as the electrolyte, CV measurement could be tested in a wide voltage window from 0–1.2 V, at different scanning rates (Figure 9a). The similar shape for all curves suggested a negligible diffusion limitation in higher scanning rates. The CV curves showed a little distortion from rectangular, especially at a high scan rate, which might be due to the porosity saturation [57] and the poor mobility of highly solvated ion Li^+^ and highly solvated anion SO_4_^2−^ [58]. The specific capacitance derived from the CV, at a scanning rate of 1 mV/s, was calculated to be 226 F/g (Figure 9c). The large specific capacitance was benefits of the interconnected structure of the NPC. The interconnected structure could provide a good electron transfer, leading to a good electrical conductivity. In addition, this interconnected structure, formed from the aggregation of the ultra-small crystal, could supply the connected pores for a continuous and efficient ion diffusion. GCD was conducted to evaluate the stability in the potential window from 0 and 1.2 V, in a 1 M Li_2_SO_4_ aqueous solution (Figure 9b). NPC exhibits almost symmetrical triangular shapes within the wide potential window, implying a reversible capacitive performance, contributed by the electric double layer supercapacitor (EDLC), and excellent coulombic efficiency. To better analyze the rate performance of the electrode materials, the GCD curves of NPC, at various current densities from 1 to 20 A/g, were collected. The specific capacitances are 205, 179, 155, 143, and 128 F/g, at current densities of 1, 2, 5, 10, and 20 A/g, respectively (Figure 9c). The maximum energy density of NPC was 10.25 Wh/kg in aqueous electrolyte, among the highest values published. The Nyquist plot of the NPC revealed that the electrical conductivity was acquired from EIS measurements. In Figure 9d, from the intersection in the Z’ axis, the interface contact resistance was as low as 0.12 Ω. The low charge transfer resistance could be revealed from the small diameter of a semicircle, which benefited from the interconnected structure. The almost vertical curve in the low-frequency region indicated a fast ion diffusion and an ideal capacitive behavior. Good cycling stability was another important factor for capacitors. The electrochemical stability of the NPC was further evaluated at a current density of 10 A/g. Figure S4 shows that there was still a 97.8% retention of capacitance, for the NPC electrodes, after 5000 cycles, confirming a long lifetime.

## 4. Conclusions

In summary, interconnected NPC was successfully prepared by direct carbonization of the ultra-small Al-MOC. With a porous structure, NPC had a high SSA (1593 m^2^/g) and a large dye adsorption, with a maximum value of 415 mg/g, proceeding the physical adsorption. The equilibrium adsorption isotherm and the adsorption kinetics have been fully discussed. The adsorption process was not influenced by the different pH values. Moreover, it was a spontaneous and endothermic process, proved by the thermodynamic studies. NPC, with an excellent adsorption capacity of MB, was believed to have excellent capacitive characteristics in supercapacitors, due to its large ion accessible area, good electrical conductivity, and suitable PSD. NPC exhibited a high specific capacitance of 226 F/g, in a symmetrical supercapacitor, suggesting a large potential in supercapacitors.

## Figures and Tables

**Table 1:** Parameters of the Langmuir and Freundlich isotherms for the adsorption of MB on the NPC samples.

	*q*_max_ (mg/g)	*K*_L_ (L/mg)	*R*_L_	*K*_F_	*n*	*R*_2_
Langmuir	417	0.317	0.0035	-	-	0.999667
Freundlich	-	-	-	220.256	8.656	0.974407

**Table 2:** Parameters of the kinetic models for the adsorption of MB on the NPC samples.

		*C*_0_ (mg/L)	
	200	400	600
*q*_e,exp_ (mg/g)	199.36	368.63	404.42
*Pseudo-1st-order*			
*q*_e_ (mg/g)	1.46	14.44	22.46
*k*_1_ (1/min)	0.01887	0.02236	0.01148
*R*_2_	0.91945	0.87247	0.90755
*Pseudo-2nd-order*			
*q*_e_ (mg/g)	199.6	369	404.9
*k*_2_ (g/mg/min)	0.000005	0.004877	0.002537
*R*_2_	0.999999	0.999996	0.999993
*Elovich*			
*α* (mg/g/min)	-	3.1 × 10^15^	5.3 × 10^17^
*β* (g/mg)	1.261	0.104	0.109
*R*_2_	0.69778	0.53520	0.80215
*Intraparticle diffusion*			
*K*_d_	0.01903	0.10303	0.58545
*C*	199.0359	366.7999	393.3279
*R*_2_	0.859991	0.949224	0.983385

**Table 3:** Thermodynamic parameters for the adsorption of MB on the NPC samples.

*C*_0_ (mg/L)	T (K)	*q*_e_ (mg/g)	*K*_D_	ΔG^θ^ (kJ/mol)	ΔH^θ^ (kJ/mol)	ΔS^θ^ (kJ/mol/K)
500	298	385	3.32	−2.98	20.28	0.078
	308	405	4.26	−3.71		
	318	417	5.05	−4.28		
	328	439	7.24	−5.40		
700	298	414	1.45	−0.92	17.69	0.062
	308	447	1.79	−1.46		
	318	475	2.11	−1.98		
	328	517	2.83	−2.84		

## Supplementary Materials

The following are available online at https://www.mdpi.com/2079-4991/9/4/601/s1, Figure S1. X-ray diffraction (XRD) pattern of Al-based metal-organic complex (Al-MOC) and the peak position of simulated MIL-53 (Al); Figure S2. Scanning Electron Microscopy (SEM) image of nanoporous carbon (NPC); Figure S3. (**a**) Transmission electron microscopy (TEM) image of NPC with mesopores pointed by arrows at the edge, (**b**,**c**) High-resolution TEM images of the thin edges of NPC with mesopores pointed by arrows; Figure S4. Specific capacitance retention for NPC at a current density of 10 A/g within 5000 cycles.
