# Supplementary material for: Evaluation of Nanoporous Carbon Synthesized from Direct Carbonization of a Metal–Organic Complex as a Highly Effective Dye Adsorbent and Supercapacitor
Source: Nanomaterials (Basel). 2019 Apr 11;9(4):601. doi: 10.3390/nano9040601 (PMC6523955; doi:10.3390/nano9040601)
Supplement: Supplementary file 1 [file nanomaterials-09-00601-s001.pdf]

Supplementary Materials: Evaluation of Nanoporous Carbon Synthesized from Direct Carbonization of Metal-organic Complex as Highly Effective Dye Adsorbent and Supercapacitor

Xiaoze Shi, Shuai Zhang, Xuecheng Chen and Ewa Mijowska

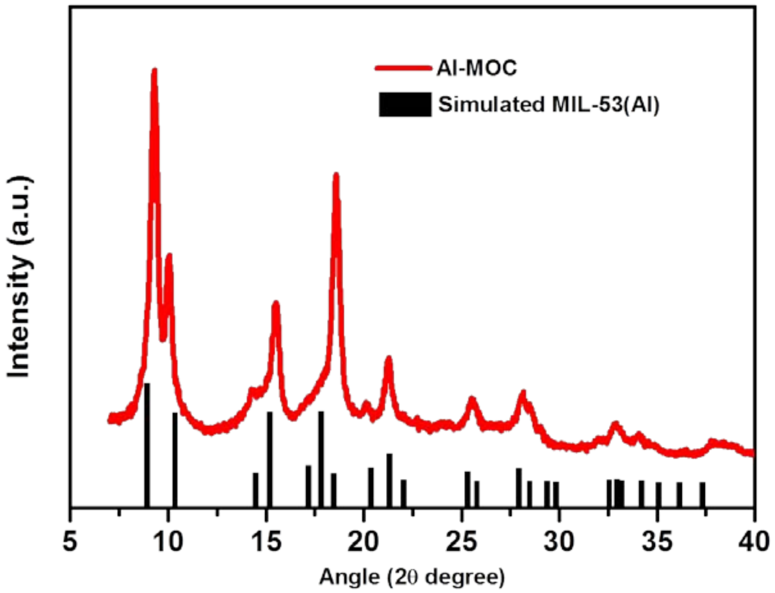

Figure 1. X-ray diffraction (XRD) pattern of Al-based metal-organic complex (Al-MOC) and the peak position of simulated MIL-53 (Al).

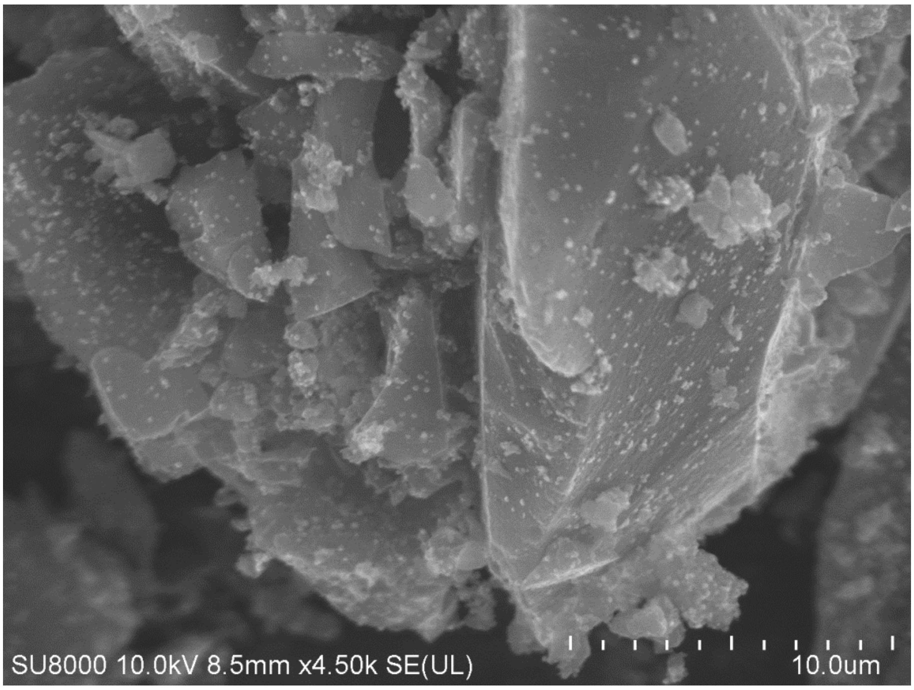

Figure 2. Scanning Electron Microscopy (SEM) image of nanoporous carbon (NPC).

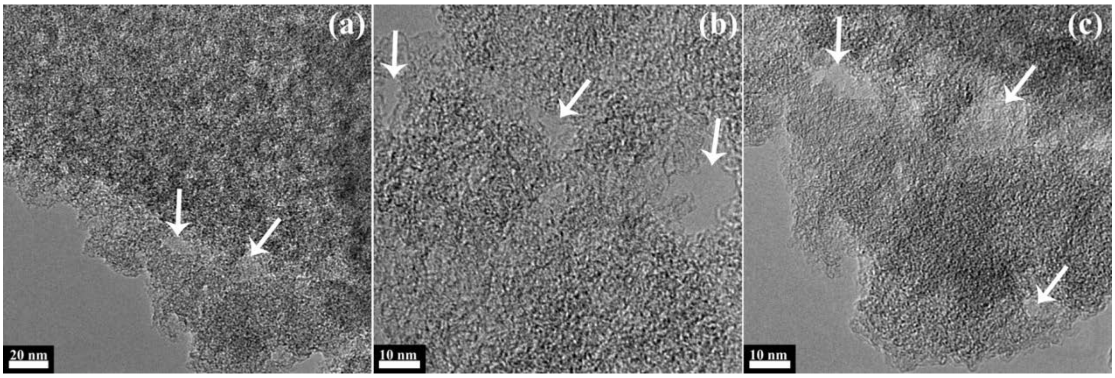

Figure 3. (**a**) Transmission electron microscopy (TEM) image of NPC with mesopores pointed by arrows at the edge. (**b**,**c**) High-resolution TEM images of the thin edges of NPC with mesopores pointed by arrows.

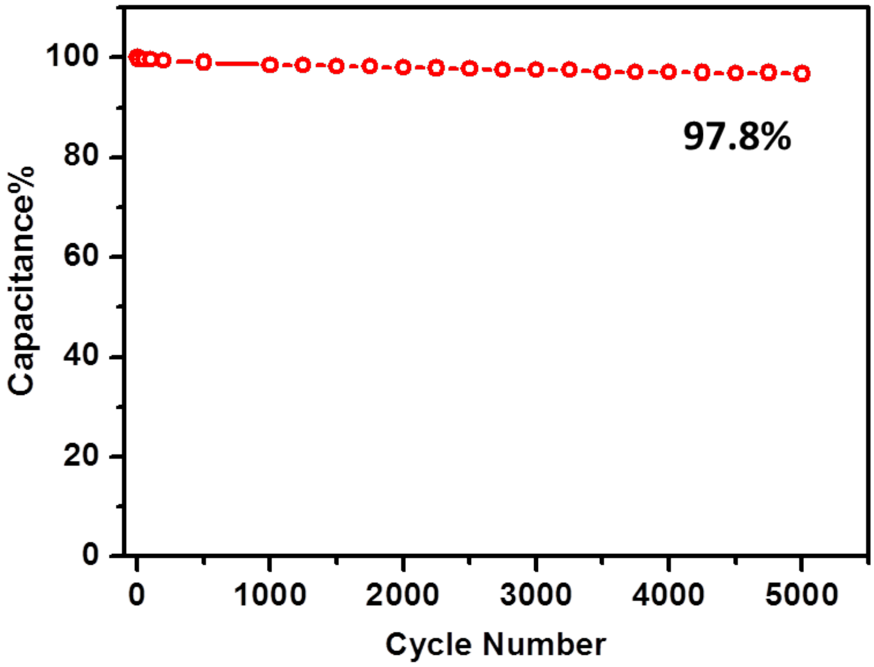

Figure 4. Specific capacitance retention for NPC at a current density of 10 A/g within 5000 cycles.
